# Leukocyte telomere length correlates with hypertrophic cardiomyopathy severity

**DOI:** 10.1038/s41598-018-29072-8

**Published:** 2018-07-25

**Authors:** Shambhabi Chatterjee, David de Gonzalo-Calvo, Anselm A. Derda, Katharina Schimmel, Kristina Sonnenschein, Udo Bavendiek, Johann Bauersachs, Christian Bär, Thomas Thum

**Affiliations:** 1 Institute of Molecular and Translational Therapeutic Strategies (IMTTS), Hannover Medical School, Hannover, Germany; 2 Institute of Biomedical Research of Barcelona (IIBB) - Spanish National Research Council (CSIC), Barcelona, Spain; 3 CIBERCV, Institute of Health Carlos III, Madrid, Spain; 4 Biomedical Research Institute Sant Pau (IIB Sant Pau), Barcelona, Spain; 5 Department of Cardiology and Angiology, Hannover Medical School, Hannover, Germany; 6 REBIRTH Excellence Cluster, Hannover Medical School, Hannover, Germany; 7 National Heart and Lung Institute, Imperial College London, London, UK

## Abstract

Telomere length is a marker of biological aging. Short leukocyte telomere length has been associated with various conditions including cardiovascular disorders. Here, we evaluated if patients with hypertrophic cardiomyopathy have altered leukocyte telomere length and whether this is associated with disease severity. A quantitative polymerase chain reaction-based method was used to measure peripheral blood leukocyte telomere length in 59 healthy control subjects and a well-characterized cohort of 88 patients diagnosed with hypertrophic cardiomyopathy: 32 patients with non-obstructive cardiomyopathy (HNCM) and 56 patients with obstructive cardiomyopathy (HOCM). We observed shorter leukocyte telomeres in both HNCM and HOCM patients compared to healthy controls. Furthermore, leukocyte telomere length was inversely associated with HCM even after adjusting for age and sex. Telomere length of HOCM patients was also inversely correlated with left ventricular outflow tract obstruction. Therefore, HOCM patients were categorized by tertiles of telomere length. Patients in the first tertile (shortest telomeres) had a significantly increased left ventricular posterior wall thickness at end-diastole and higher left ventricular outflow tract gradients, whereas the left ventricular end-diastolic diameter was lower compared with patients in the second and third tertile. In summary, telomere length is associated with the severity of the disease in the HOCM subtype.

## Introduction

Hypertrophic cardiomyopathy (HCM) is a complex heart disease which is most commonly caused by a single mutation in genes encoding for sarcomeric protein. To date more than 1400 different mutations in at least 11 genes have been identified affecting approximately one in five hundred individuals of the general population^1^. Interestingly, in many individuals the disease is relatively asymptomatic and does not necessarily reduce the quality of life. In stark contrast, HCM can have fatal consequences since it is a very common cause of sudden cardiac death^2^. Pathophysiologically, HCM is characterized by left ventricular hypertrophy, adverse cardiac remodeling, fibrosis, atrial fibrillation, and may ultimately culminate in heart failure^3^. HCM can be sub-classified as hypertrophic obstructive cardiomyopathy (HOCM) and hypertrophic non-obstructive cardiomyopathy (HNCM). The key criterion for the classification of HOCM is a pathological left ventricular outflow tract (LVOT) obstruction caused by asymmetric hypertrophy of the septum^4^. Despite the clinical relevance and prevalence of the disease, there is a lack of non-invasive biomarkers that can facilitate the management of HCM and their subtypes. Thus, novel approaches seem fundamental.

Telomeres are heterochromatic stretches of repetitive DNA at the ends of linear chromosomes which are essential for a cell’s evasion of illegitimate DNA repair or for the protection against telomere shortening by exonucleolytic DNA degradation^5^. Nevertheless, telomeres shorten naturally owing to a phenomenon known as “end replication problem”, which describes the loss of telomeric DNA upon each cell division due to the failure of replicative DNA polymerases to faithfully replicate linear DNA ends^6^. Telomeres are inherited and consequently, exhibits sequential reduction with increase in age and can reflect the proliferation history of a given cell or tissue. Of note, the telomere length can differ even amongst age-matched individuals due to different rates of telomere attrition as a result of multiple intrinsic and extrinsic factors^7^. Along these lines, peripheral blood leukocyte telomere length (LTL) has been proposed as a biomarker of biological ageing^7,8^. Cellular senescence, oxidative stress, lifestyle are some of the key factors which determine changes in telomere lengths. The level of stress has been reported to have a strong impact on telomere lengths as well^9^. Stress is a notable risk factor for several age related diseases, including heart failure. Since shortening of telomere lengths is influenced by stress, one can speculate a compelling link between age-associated disorders and telomere length. Short LTL has been associated with several pathological conditions affecting variety of organ systems in the human body such as bone marrow causing aplastic anemia^10^, competency of the immune system^11^, type 2 diabetes^12^, or childhood autism^13^. The study performed in patients with ischemic cardiomyopathy reported shorter telomere length in the circulating leukocytes of patients which correlated well with telomere length and functionality of their bone-marrow^14^. Furthermore, short LTL is a potential prognostic biomarker and has been explored in a number of heart conditions^7,15–18^. The ease of access to blood samples and circulating leukocytes makes LTL a promising candidate for biomarkers. Nevertheless, to our knowledge the relationship between LTL and a hereditary cardiac disease such as HCM has not been investigated to date. To fill this gap we used our well-characterized cohort of 88 patients diagnosed with hypertrophic cardiomyopathy^19,20^ and compared their LTL with those of healthy individuals (n = 59). Furthermore, we analyzed potential differences in LTL comparing HNCM and HOCM patients.

## Results

### LTL inversely correlates with age and is shorter in patients with HCM

**Figure 1:**
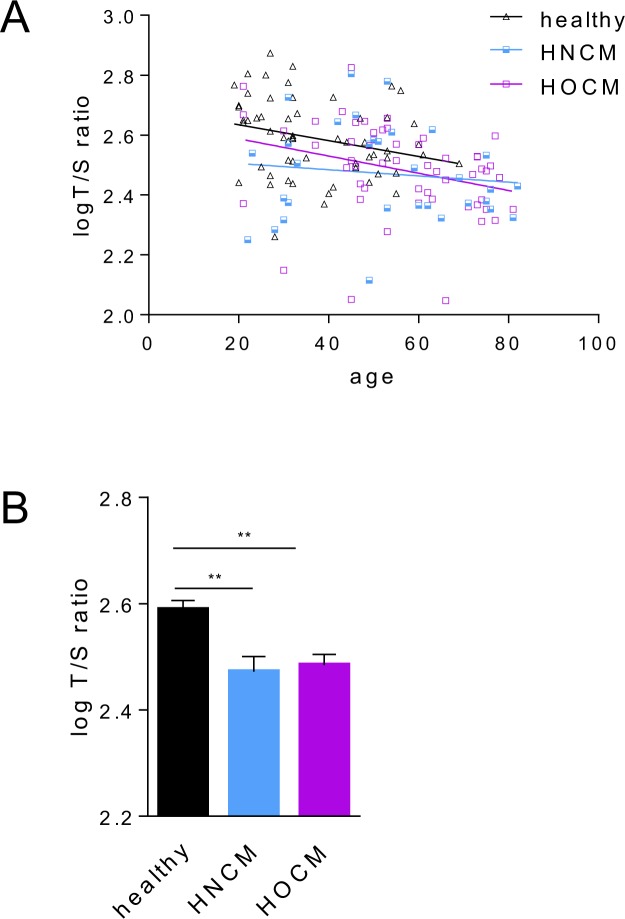
Telomere length distribution in control, HNCM and HOCM. (**A**) Relative leukocyte telomere length expressed as the log-transformed ratio of the telomeric sequence to single reference gene (T/S), plotted as a function of age. The negative slopes of the linear regression lines indicate age-related telomere shortening. (**B**) Average log-transformed T/S ratio in control subjects versus HNCM and HOCM patients. The results are shown as mean ± SD. Between-group differences were analyzed using one-way ANOVA, followed by Tukey’s post hoc test. **P < 0.010.

We first measured LTL in blood samples from 59 healthy subjects (37.1 y, age range: 19.0-69-0 y; 35 male and 24 female) and 88 patients with HCM. Within the HCM cohort 32 patients were diagnosed with HNCM (age range: 22.0–82.0 y; 23 male and 9 female) and 56 patients with HOCM (age range: 21.0–81.0 y; 20 male and 36 female), based on diagnostic criteria described in the methods section. Detailed patient characteristics echocardiography data, clinical symptoms and drug regimens are described in Table 1. As expected, LTL expressed as the ratio of the telomeric sequence to single reference gene (T/S)^21^ was inversely correlated with age in healthy subjects and both HCM patient groups (Fig. 1A). Average LTL was significantly higher in healthy controls compared to both patient groups, HOCM and HNCM (Fig. 1B). Using an ANCOVA model to control for the confounding effect of age, this difference remained statistically significant (*P* < 0.050 for both comparisons) (Supplemental Table 1). No differences in average LTL between both HCM subtypes were observed (Fig. 1B). Since both patient groups showed similar LTL, we explored in detail the association between leukocyte telomere length and HCM using logistic regression models. HCM was entered as a dependent variable and subsequently T/S was entered as independent variable (model 1). Model 1 was also adjusted for age (model 2) and age and sex (model 3) in order to take in to account possible confounders associated with LTL. Supporting our previous findings, we observed an inverse association between leukocyte telomere length and HCM, even after adjusting for age and sex (Table 2). To further corroborate these results, we performed an additional sub-analysis with strictly age- and sex- matched healthy controls and HCM patients. The results supported our previous findings (Supplemental Table 2). In summary, shorter LTL is a distinct signature of HCM patients compared to healthy subjects independent of potential confounders, such as age and sex, without any difference in LTL between the two subtypes of the disease, HOCM and HNCM.

**Table 1:** Characteristics of the study population.

Variable	HCM	HNCM	HOCM	*P*-value
	N = 88	N = 32	N = 56	
Age (years)	54.5 ± 16.6	52.0 ± 18.2	55.9 ± 15.5	0.425
Male N (%)	43 (49)	23 (72)	20 (36)	0.002
Body mass index (kg/m^2^)	22.1 ± 11.6	21.6 ± 11.6	22.4 ± 11.7	0.651
**Echocardiography**				
IVS (mm)	20.8 ± 8.1	19.3 ± 6.9	21.6 ± 8.6	0.153
LVEDD (mm)	43.0 ± 6.0	44.3 ± 5.8	42.2 ± 6.1	0.136
Aortic root (mm)	31.5 ± 4.6	31.4 ± 4.6	31.5 ± 4.7	0.826
LVPWD (mm)	11.7 ± 3.5	11.1 ± 2.8	12.2 ± 3.9	0.256
LVOT-gr.max. (mmHg)	73.1 ± 61.2	12.5 ± 10.1	90.4 ± 58.6	<0.001
LVEF reduced N (%)	9 (10)	9 (28)	0 (0)	<0.001
RVEF reduced N (%)	8 (9)	7 (22)	1 (2)	0.004
Mitral insufficiency N (%)				
minor	47 (53)	22 (69)	25 (45)	0.067
medium	29 (33)	7 (22)	22 (39)	0.095
major	5 (6)	1 (3)	4 (7)	0.645
missing	7 (8)	2 (6)	5 (9)	
**Clinical symptoms**				
Syncope N (%)	14 (16)	3 (9)	11 (20)	0.230
Positive family history N (%)	25 (28)	6 (19)	19 (34)	0.134
Dyspnoea N (%)	46 (52)	14 (44)	32 (57)	0.163
NYHA N (%)				0.223
1	21 (24)	11 (34)	10 (18)	
2	37 (42)	10 (31)	27 (48)	
3	18 (21)	6 (20)	12 (21)	
4	1 (1)	0 (0)	1 (2)	
missing	11 (12)	5 (15)	6 (11)	
Angina pectoris N (%)	13 (15)	2 (6)	11 (20)	0.116
Palpitations N (%)	26 (30)	9 (28)	17 (30)	0.808
Peripheral edema N (%)	10 (11)	4 (13)	6 (11)	1.000
Hypertension N (%)	30 (34)	12 (38)	18 (32)	0.640
Diabetes mellitus N (%)	8 (9)	3 (9)	5 (9)	1.000
Coronary artery disease N (%)	13 (15)	4 (13)	9 (16)	0.759
**Drugs**				
Beta blockers N (%)	65 (74)	24 (75)	41 (73)	1.000
ACE inhibitors N (%)	27 (31)	9 (28)	18 (32)	0.806
AT1 antagonists (%)	7 (8)	6 (19)	1 (2)	0.009
Diuretics N (%)	29 (33)	10 (31)	19 (34)	0.812
Calcium antagonists N (%)	17 (19)	6 (19)	11 (20)	1.000
Anticoagulation drugs N (%)	38 (43)	13 (41)	25 (45)	0.816

Data are presented as mean ± SD for continuous variables and as frequencies (percentages) for categorical variables.

IVS = interventricular septum size; LVEDD = left ventricular end-diastolic diameter; LVPWD = left ventricular posterior wall thickness end diastole; LVOT-gr. max. = left ventricular outflow tract gradient maximum; LVEF = left ventricle ejection fraction; RVEF = right ventricle ejection fraction; ACE: Angiotensin-converting enzyme; AT1 = angiotensin I.

**Table 2:** Association between leukocyte telomere length and HCM.

	OR (95% IC)	*P*-value
Model 1		
T/S	0.004 (0.001, 0.060)	<0.001
Model 2		
T/S	0.019 (0.001, 0.260)	0.003
Age	1.066 (1.037, 1.096)	<0.001
Model 3		
T/S	0.030 (0.002, 0.496)	0.014
Age	1.068 (1.039, 1.098)	<0.001
Sex	0.687 (0.313, 1.501)	0.351

OR: Odds Ratio, CI: Confidence Interval.

### Leukocyte telomere length is associated with the severity of HOCM

**Figure 2:**
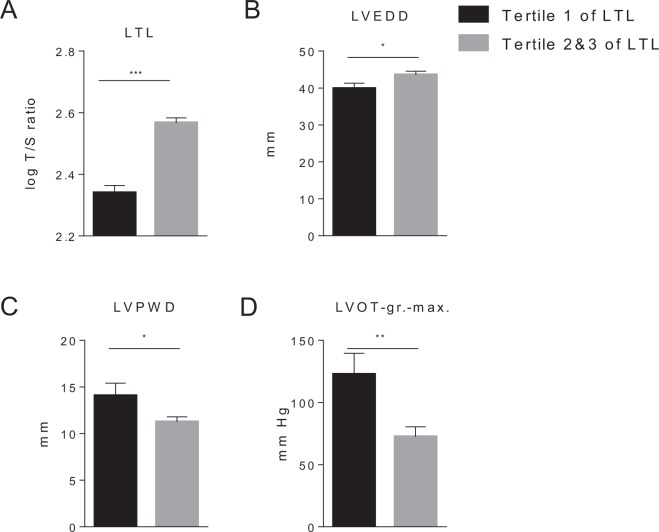
LTL is negatively associated with HOCM severity. HOCM patients were stratified by tertiles of telomere length. (**A**) Patients in the first tertile demonstrated significantly higher LVOT grad. max (**D**) and LVPWD (**C**) values, while LVEDD (**B**) was significantly lower in comparison to patients in tertile 2 & 3. The results are shown as mean ± SD. Between-group differences were analyzed using Student’s t-test for independent samples. *P < 0.050, **P < 0.01, ***P < 0.001.

We performed correlation analyses to explore the potential association of LTL with different echocardiographic parameters that are characteristic for HNCM and HOCM. As shown in Table 3, we observed a direct correlation between LTL and the left ventricular end-diastolic diameter (LVEDD) in patients with HOCM (r = 0.281, *P* = 0.044). Interestingly, an inverse association between LTL and the LOVT gradient maximum (LVOT-gr.max) was observed in the same patients (r = −0.291, *P* = 0.043). No correlation was observed between LTL and any echocardiographic parameter in the HNCM group (data not shown). Since LVOT-gr.max is one of the key clinical parameters to distinguish HNCM from HOCM^19^, we performed additional analysis to further explore this correlation in detail. To this end, we analyzed the same echocardiographic parameters (as in Table 3) in those HOCM patients in the first tertile of LTL and those patients in the second and third tertiles of LTL. Strikingly, we observed significantly higher levels of LVOT-gr.max in those patients in the first tertile of LTL (*P* = 0.004) (Fig. 2). Similarly, we observed significantly lower levels of LVEDD and higher levels of LVPWD in this group (*P* < 0.050 for both comparisons) (Fig. 2). Similar findings were observed when we compared participants in tertile 1 and tertile 3 (Supplemental Fig. 1). No differences in the prevalence of potential confounding factors such as hypertension, diabetes mellitus and coronary artery disease were observed between study groups (*P* > 0.050 for all comparisons). Finally, since different causative mutations for HCM may have an impact on disease severity, we analyzed the most commonly affected genes *Myosin Heavy Chain* (*MYH7*) and *Myosin Binding Protein C, Cardiac* (*MYBPC3*) in a subset of patients who gave consent for genetic testing (N = 22, 25%)^19^. No differences were observed in the prevalence of the mutations in *MYH7* and *MYBPC3* between HNCM and HOCM patients (*P*-value = 0.203), which is in line with a recent study demonstrating that the clinical phenotype is independent of gene mutation and mutation dosage^22^. Similarly, no differences were observed in the prevalence of these between HOCM patients in tertile 1 and tertiles 2 and 3 of telomere length (*P*-value = 0.198).

**Table 3:** Correlation between leukocyte telomere length and echocardiographic parameter.

		IVSED	LVEDD	LVPWD	LVOT-gr.max
HNCM	r	0.084	−0.087	0.042	−0.101
	*P*-value	0.654	0.641	0.824	0.731
HOCM	r	0.165	0.281	−0.170	−0.291
	*P*-value	0.238	0.044	0.265	0.043

Taken together, LTL is negatively associated with LVOT-gr.max in HOCM patients, thus LTL may be indicative of the severity of HOCM.

## Discussion

This is the first study to investigate circulating LTL in a cohort of HCM patients. The major findings are that LTL is significantly shorter in patients with HCM, and secondly, in the HOCM sub-population LTL negatively associates with the severity of the disease. In contrast, LTL is not different between the obstructive and non-obstructive forms of HCM.

Interestingly, Sharifi-Sanjani and colleagues recently reported that shorter telomere length is a cardiomyocyte-specific feature of end-stage failing hearts including those of HCM patients^23^. LTL shortening most likely reflects the proliferation history of the bone marrow while telomere attrition in post-mitotic cardiomyocyte is presumably caused by other factors such as oxidative stress and DNA damage^24,25^. Nevertheless, a limitation of both our and their study is the investigation of telomere lengths at only a single time point (end-stage heart failure) and, therefore, it remains unclear whether HCM cardiomyocytes start off with shorter telomeres or whether they shorten during the progression of the disease. Nevertheless, both studies undoubtedly link shorter telomere length to HCM. Further longitudinal studies, e.g. through periodic blood sampling or side-by-side analysis of the same heart after myectomy and explantation, are warranted to elucidate the causality of shorter telomeres in HCM.

We also explored a potential correlation of LTL with disease characteristics as determined by echocardiography. While there was no correlation in the HNCM cohort between LTL and echocardiography parameters, LTL significantly correlated with echocardiography parameters characteristic for HOCM (i.e. LVOT grad. max). Further analyses revealed that the HOCM patients with the shortest LTL (1^st^ tertile) had significantly higher LVOT grad. max, LVPWD and lower LVEDD (compared to 2^nd^ and 3^rd^ tertiles). Thus, LTL in HOCM patients may serve as an indicator of disease severity. Because echocardiography characterization is required to separate HOCM from HNCM cases, this may raise the question about the clinical use of LTL. However, based on our findings, further studies are warranted to extend the association studies beyond echocardiography parameters in order to investigate for instance the prognostic value of LTL in HOCM patients. Of note, LTL measurements require only small amounts of peripheral blood and are relatively inexpensive and effortless. Future inclusion of LTL measurements upon diagnosis of HOCM will therefore be crucial to further our understanding of the mechanisms and the significance of the association between LTL and HOCM severity, which will potentially improve clinical decision-making. In support of this, in a large study of hypertensive patients with left-ventricular hypertrophy LTL was associated with ischemic heart disease and reduced LTL was predictive of cardiovascular disease during a follow-up period^26^. However, two large population-based studies revealed that longer LTL are positively associated with left ventricular mass in both hypertensive and normotensive individuals^27,28^. In contrast, the present study identified reduced LTL as a specific signature of HCM and may therefore point toward genetic basis for this association.

LTL (T/S ratio) plotted as a function of the patients’ age showed an expected age-related loss of telomere length in all study groups (HNCM, HOCM and healthy). Several studies used the slope of the linear regression line to express the yearly rate of telomere attrition^8,9,17^. Shorter average LTL in HCM patients compared to controls may be attributed to inherited shorter LTL or could be caused by accelerated telomere attrition that is associated with the disease itself. In the latter scenario, one would expect a significantly steeper slope for HCM compared to the healthy group, a difference which we did not observe. While this may point toward a hereditary component, in the absence of longitudinal data and knowledge on confounding factors which may affect LTL shortening, we avoid drawing such conclusions here. The average telomere length in adult healthy human cardiomyocytes are relatively stable but exhibit shortening during ageing and heart disease^29^. In this regard, telomere shortening can be affected by genetic factors such as rapid turnover of hematopoietic stem/progenitor cells or hereditary predisposition. Critically short telomeres induce mitochondrial dysfunction which further puts the heart at risk and impedes cardiac function^30^. However, chronic inflammation, oxidative stress in combination with high turnover of white blood cells are likely the most compelling factors leading to loss of leukocyte telomeres in patients suffering from HCM similar to coronary heart disease patients^31,32^.

Strengths of our study are that we not only compared healthy subjects with HCM patients but also the well phenotyped HCM cohort which allowed us to discriminate between HNCM and HOCM to further identify a correlation between LTL and HCM severity. Moreover, the latter was identified in a sub-cohort of 56 patients, thus allowing robust statistical evaluation. Nevertheless, some limitations of the present study should be noted. First, although the qPCR-based protocol for telomere measurements is widely accepted and the most frequently applied method in larger cohort studies, it measures mean telomere length but not individual short telomeres. In this regard, telomere dysfunction is determined by the frequency of critically short telomeres and the rate of increase of short telomeres predicts longevity in mammals^33^ and may potentially be associated with disease. Therefore, more sensitive protocols such as HT-qFISH^8^ or Flow-FISH^34^ allowing the assessment of critically short telomeres in individual cells should be considered in follow-up studies. Second, despite the robustness of the associations observed here, larger sample sizes for all sub-cohorts in future validation studies are desirable. Third, due to the lack of serial blood sampling (i.e. long term follow-up) our study design does not allow to determine whether the observed associations imply causal relationships, and fourth, does not allow to identify a correlation between telomere length in myocardium and leukocytes. Fifth, there is a significant difference in the age range of healthy controls and HCM patients. Nevertheless, re-analysis after strict age and sex matching of subpopulations showed no difference compared to entire cohorts. Finally, information on the underlying gene mutation was available for only 25% of all patients, thus reliable correlation of LTL with different HCM causative mutations was not possible.

In conclusion, this is the first study to reveal short LTL as a general signature of HCM patients. Intriguingly, LTL correlates with disease severity in HOCM which strongly warrants investigation in larger cohorts utilizing more sensitive LTL measurement method to further validate LTL as a clinically relevant biomarker in HCM.

## Methods

### Patient data and Control samples

Enrolled patients were selected of those who were subjected to the Hannover Medical School outpatient clinic because of unclear cardiac hypertrophy. Out of those, all patients clinically defined as HNCM and HOCM patients were included (see clinical parameters below). Blood was sampled in EDTA containing tubes using standardized protocols and kept frozen (−80 °C) until DNA isolation of all samples (healthy and HCM). Blood collection of patients took place between 2011 and 2016, while blood of healthy controls was collected in 2012–2016 from the Hannover Medical School blood donation service. We obtained written informed consent from all patients and the study was approved by the local ethical committee of Hannover Medical School. The study was performed in accordance with the guidelines from the declaration of Helsinki and its amendments or comparable ethical standards. The patients were diagnosed depending upon the presence of a hypertrophic cardiac septum ≥15 mm or ≥13 mm in case of ECG abnormalities and/or positive family history. These parameters were chosen for diagnosis according to the recently established European guidelines for the diagnosis and management of hypertrophic cardiomyopathies^3^. The peak Doppler LV outflow tract gradient of ≥30 mm Hg was considered to be left ventricular outflow tract obstruction.

### DNA isolation

Genomic DNA for telomere measurements was isolated according to standard procedures from 50 µL blood using the DNeasy Blood & Tissue Kit (Qiagen #69506) and stored at −20 °C. DNA samples were diluted in 96-well plates to a fixed concentration of 10 ng/µl.

Mutation analysis of the *MYBPC3* and *MYH7* genes was done on isolated genomic DNA as previously described^19^.

### Real-time PCR-based LTL measurement

A quantitative polymerase chain reaction (qPCR)-based assay was performed to measure the relative telomere length. The method compares mean telomere repeat sequence (TL) to a reference single copy gene (*36B4*) following the same principle as previously described^21^ but with several optimizations of the experimental settings^35^. Each DNA sample was run on the qPCR as triplicates for both the telomere primer and *36B4* primer. One DNA sample with long (DNA from human induced pluripotent stem cells, passage 45) and another with short telomeres (DNA from human umbilical vein endothelial cells, passage 5) were used as inter run calibrators for each qPCR run. A standard curve was generated from serially-diluted reference genomic DNA of K562 cell line for each of the qPCR runs.

Following primers were used in this study:

**Table:** 

Human TL FW	GGTTTTTGAGGGTGAGGGTGAGGGTGAGGGTGAGGGT
Human TL RV	TCCCGACTATCCCTATCCCTATCCCTATCCCTACCCTA
Human *36B4* FW	CAGCAAGTGGGAAGGTGTAATCC
Human *36B4* RV	CCCATTCTATCATCAACGGGTACAA

The qPCR runs performed on a Viia7 Real-Time PCR system (Thermo Fisher Scientific) were analyzed for T/S ratio as previously described^35^.

### Statistical analysis

Due to the lack of previous data or similar studies evaluating T/S ratios in HCM no *a priori* calculation of the appropriate sample size was performed. However, a post hoc power calculation accepting an alpha risk of 0.05 in a two-sided test with 59 subjects in the healthy group and 88 in the HCM group, the statistical power was 100% to recognize a statistically significant difference of means between both groups.

Data are presented as mean ± SD for continuous variables and as frequencies (percentages) for categorical variables. Data normality was evaluated using the Kolmogorov-Smirnov test. Categorical variables were compared between groups using Fisher’s exact test. Non-normally distributed variables, including T/S ratio, were logarithmically transformed to account for nonlinearity. Continuous variables were compared between the groups using Student’s t-test for independent samples and one-way ANOVA, followed by Tukey’s post hoc test, for comparisons between each subgroup. LTL was also adjusted for age with ANCOVA models to account for differences between study groups. Correlations between variables were analyzed using Pearson’s correlation analysis and the results presented using Pearson’s correlation coefficient (r). Logistic regressions were analyzed to explore the association between LTL and HCM. HCM was entered as a dependent variable and subsequently, LTL was entered as independent variable (model 1). In order to establish whether the observed association between HCM and LTL could be influenced by possible confounders, model 1 was also adjusted for age (model 2) and age + sex (model 3). The results are presented as the odds ratio (OR) and 95% confidence intervals (CI). Values of *P* < 0.05 were considered statistically significant. All statistical analyses were performed using the statistical software R (www.r-project.org).

### Data availability

All data generated or analyzed during this study are included in this published article (and its Supplementary Information files).

## Electronic supplementary material

Supplemental Information
