# Supplementary material for: Leukocyte telomere length correlates with hypertrophic cardiomyopathy severity
Source: Sci Rep. 2018 Jul 25;8:11227. doi: 10.1038/s41598-018-29072-8 (PMC6060137; doi:10.1038/s41598-018-29072-8)
Supplement: Supplementary file 1 — Supplemental Information [file 41598_2018_29072_MOESM1_ESM.pdf]

Leukocyte telomere length correlates with hypertrophic cardiomyopathy severity

Shambhabi Chatterjee[†], David de Gonzalo-Calvo[†], Anselm A. Derda, Katharina Schimmel, Kristina Sonnenschein, Udo Bavendiek, Johann Bauersachs, Christian Bär[*] and Thomas Thum[*]

SUPPLEMENTAL INFORMATION

*Corresponding Authors

[†] equal contribution

Supplemental Table 1. Leukocyte telomere levels in study groups (adjusted by age)

Variable	Healthy	HNCM	HOCM
**logT/S ratio**	2.57 ± 0.02	2.48 ± 0.03	2.47 ± 0.2 $^{b\ cc}$

Data are presented as estimated marginal mean ± SEM.

Healthy *vs.* HNCM: b < 0.050. Healthy *vs.* HOCM: cc < 0.010.

Supplemental Table 2. Strictly age- and sex- matched healthy controls and HCM patients.

Variable	Healthy	HCM	P-value
N	30	30	
Age (years)	42.5 ± 14.0	41.6 ± 14.0	0.913
Male N (%)	16 (53)	16 (53)	
logT/S ratio	2.57 ± 0.11	2.49 ± 0.17	0.040

Data are presented as mean ± SD for continuous variables and as frequencies (percentages) for categorical variables.

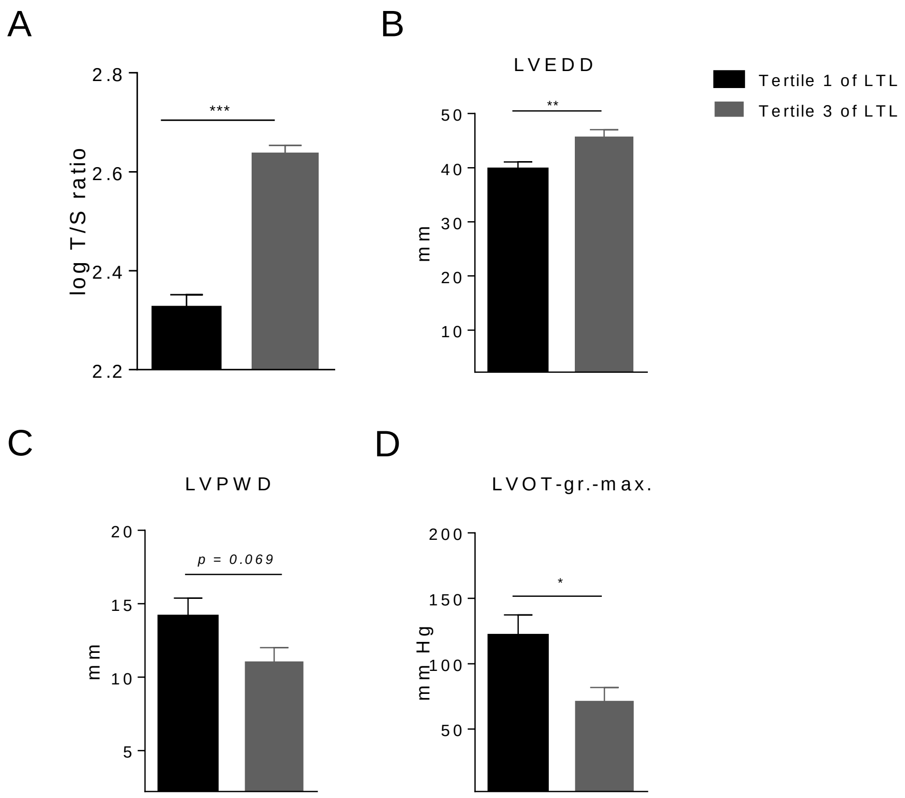

Supplemental Figure 1| LTL is negatively associated with HOCM severity. HOCM patients were stratified by tertiles of telomere length (A). Patients in the first tertile demonstrated significantly higher LVOT grad. max (D) and LVPWD (C) values, while LVEDD (B) was significantly lower in comparison to patients in tertile 3. The results are shown as mean ± SD. Between-group differences were analyzed using Student's t-test for independent samples. *: $P < 0.05$, ***: $P < 0.001$.
